# Superficial Parasternal Intercostal Plane Block and Full Sternotomy; A Randomized Trial

**DOI:** 10.1093/ejcts/ezaf226

**Published:** 2025-07-08

**Authors:** Antti Kalli, Julia Vistbacka, Eeva Moilanen, Kati Järvelä, Ari Mennander

**Affiliations:** Faculty of Medicine and Health Technology, Tampere University, Tampere 33520, Finland; Tampere University Hospital, Heart Hospital, Tampere 33520, Finland; The Immunopharmacology Research Group, Faculty of Medicine and Health Technology, Tampere University and Tampere University Hospital, Tampere 33520, Finland; The Immunopharmacology Research Group, Faculty of Medicine and Health Technology, Tampere University and Tampere University Hospital, Tampere 33520, Finland; Faculty of Medicine and Health Technology, Tampere University, Tampere 33520, Finland; Tampere University Hospital, Heart Hospital, Tampere 33520, Finland; Faculty of Medicine and Health Technology, Tampere University, Tampere 33520, Finland; Tampere University Hospital, Heart Hospital, Tampere 33520, Finland

**Keywords:** aortic valve replacement, SPIP, lung function, interleukin-6

## Abstract

**Objectives:**

Cardiac surgery via full sternotomy impacts postoperative lung function. We studied whether ultrasound-guided superficial parasternal intercostal plane block (SPIP) before surgical aortic valve replacement via full sternotomy would ameliorate postoperative lung function and filtration capacity.

**Methods:**

A total of 74 consecutive patients undergoing surgical aortic valve replacement were randomized to receive either or not additional SPIP. Pre- and postoperative lung function tests were compared among the patients. Venous and arterial blood samples were collected to calculate lung filtration (venous/arterial) of the inflammatory factors chemerin, chitinase-3-like protein 1 (YKL-40), resistin, and interleukin-6 (IL6) immediately before (T1), 1 hour after releasing aortic cross-clamp (T2), and on the following morning (T3) after surgery in 30 age- and sex-adjusted patients.

**Results:**

Patients with SPIP were older as compared to those without (66.7 [10.7] vs 60.2 [13.4], years, respectively, *P* < 0.04). Neither other patient characteristics nor preoperative lung functions differed between the patient groups. Forced expiratory volume in 1 second (FEV), forced volume capacity (FVC), and relative FVC changes decreased less in patients treated with wound analgesia as compared to those without (*P *= 0.024, *P *= 0.042, and *P *= 0.042). Total oxycodone consumption (*P *= 0.634), YKL-40, and resistin did not differ between the groups. Arterial chemerin decreased and venous/arterial IL6 ratio increased in patients with SPIP as compared to those without (*P *= 0.024 versus *P* = 0.332, respectfully).

**Conclusions:**

SPIP before aortic valve surgery via full sternotomy impacts postoperative respiratory function and venous/arterial IL6 ratio.

**Clinical registration number:**

The study was approved by the institutional review board (Ethical Committee of the Tampere University Hospital, Tampere, Finland, registration number R18011M) on March 8, 2018, and the study conforms to the ethical guidelines of the Declaration of Helsinki. The trial was registered as ClinicalTrials.gov ID NCT03704753 (EudraCT = 2017-004744-38).

## Graphical abstract

**Figure:**
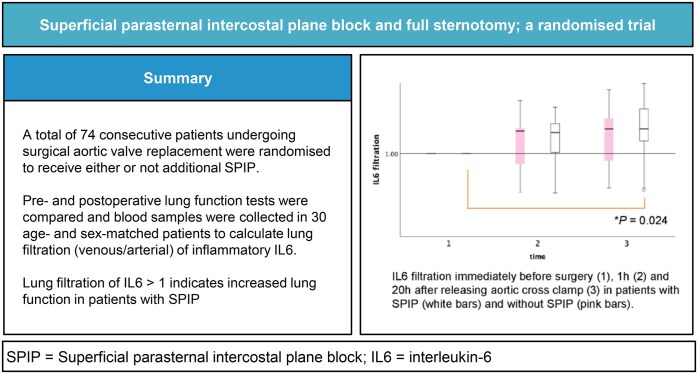

## INTRODUCTION

Cardiac surgery and use of cardiopulmonary bypass represent together a major inflammatory trauma necessitating postoperative pain management.^1^^,^^2^ Pain management is essential after major surgery,^3–5^ and adjunct local anaesthesia may enhance recovery.^6–14^ Early patient recovery after cardiac surgery via full sternotomy is dependent on postoperative pulmonary function. Wound analgesia together with pain management may thus facilitate early recovery and enhance lung function and filtration capacity after cardiac surgery via full sternotomy. Filtration capacity is an innovating concept referring to the efficacy of eliminating or producing inflammatory agents during passage through the lungs.^15^^,^^16^

Only few previous studies have investigated preoperative and postoperative lung function tests in patients undergoing cardiac surgery with and without additional wound analgesia.^17^^,^^18^ Laboratory markers reflecting postoperative pain control via enhanced lung function capacity would offer means to estimate early patient recovery after cardiac surgery. Sensitive markers of inflammation such as serum chemerin, chitinase-3-like protein 1 (YKL-40), resistin, and interleukin-6 (IL6) may reveal the inflammatory response after cardiac surgery depending on the lung function capacity.^19^^,^^20^

Chemerin and resistin are proinflammatory adipokines found in perivascular adipose tissues and lungs.^20–22^ Interestingly, chemerin has been associated with the development of both clinical and experimentally induced pulmonary hypertension.^23–25^ Resistin increases vascular remodelling, immune cell recruitment, and proinflammatory IL-6 production.^20^ YKL-40 is a sensitive marker of inflammation after surgical trauma^26^ and in the respiratory tract.^19^

We hypothesized that wound analgesia using ultrasound-guided superficial parasternal intercostal plane block (SPIP) before aortic valve surgery via full sternotomy ameliorates postoperative lung function reflecting changes in some inflammatory laboratory markers. In addition, we investigated the venous and arterial concentrations of the inflammatory factors chemerin, YKL-40, resistin, and IL6 in patients with and without SPIP to estimate their elimination/production during passage through the lungs.

## METHODS

### Ethical statement and study design

The CONSORT Reporting Protocol was applied. The trial was registered as ClinicalTrials.gov ID NCT03704753 (EudraCT = 2017-004744-38). Briefly, 74 consecutive and elective patients undergoing primary aortic valve replacement were enrolled in the study after careful attention on exclusion and inclusion criteria between August 2019 and February 2024 (Supplementary File).

### Data availability

The data in this study will be provided upon reasonable request to the corresponding author after approval from the Tampere University Hospital Ethics Committee.

### Preoperative wound analgesia

The patients were randomized to either having preoperative SPIP using 40 mL of ropivacaine (7.5 mg/mL, Fresenius Kabi AB, Uppsala, Sweden) or saline (9 mg/mL, Fresenius Kabi AB, Uppsala, Sweden) (Supplementary File).

### Anaesthesia and surgery

The surgical technique encompassed single aortic valve replacement through full median sternotomy while using continuous infusion of propofol and remifentanil. Pleurostomy was case-selective (Supplementary File).

### Preoperative and postoperative respiratory function

Respiratory lung functions were evaluated prior and the following day after surgery after surgery by spirometry (Care Fusion Micro I, CareFusion USA). The following parameters were evaluated and calculated using the highest out of 3 values for each patient: forced expiratory volume in 1 second (FEV), forced vital capacity (FVC), peak expiratory flow (PEF), preoperative FEV minus postoperative FEV (FEV change), preoperative FVC minus postoperative FVC (FVC change), preoperative PEF minus postoperative PEF (PEF change), preoperative FEV minus postoperative FEV divided by preoperative FEV (Relative FEV change), preoperative FVC minus postoperative FVC divided by preoperative FEV (Relative FVC change), and preoperative PEF minus postoperative PEF divided by preoperative PEF (Relative PEF change).

### Sample collection and ELISA measurement of chemerin, YKL-40, resistin, and IL6

Arterial and venous blood samples were collected immediately after induction of anaesthesia (T1), 1 hour after releasing aortic cross-clamp (T2), and the following day after surgery at 20 hours after releasing aortic cross-clamp (T3) using simultaneously taken blood samples from the radial artery and the superior vena cava, respectively. The time points were selected according to the early response of inflammatory markers in patients undergoing cardiopulmonary bypass.^16^ To further minimize possible effect of age and sex on lung volume function, the samples were evaluated from 30 age- and sex- adjusted patients with and without wound analgesia at every time point mentioned. Chemerin, YKL-40, resistin, and IL6 levels in plasma samples were determined by enzyme-linked immunosorbent assay (ELISA) using commercial reagents from Invitrogen/Thermo Fischer Scientific, Vienna, Austria (IL6) and R&D Systems Europe, Abingdon, UK (the others). The detection limits and inter-assay coefficients of variation were 7.8 pg/mL and 6.1% for chemerin, 15.6 pg/mL and 6.0% for YKL-40, 7.8 pg/mL and 6.9% for resistin, and 2 pg/mL and 6.8% for IL6.

### Statistical analysis

The included patients were assigned to receive either SPIP or not using a computer-based randomization (Supplementary File). The study sample size was estimated according to a previous study by Barr *et al.*,^27^ a study on parasternal intercostal block for pain management after cardiac surgery. Using a 2-sided alpha of 0.05 and an at least 8 mg difference in oxycodone consumption, statistical power of 80% needed at least 52 patients.

Continuous variables were expressed as means with standard deviation and compared using the Mann-Whitney *U*-test. Categorical variables were presented as numbers and percentages and were compared using Fisher’s exact test. Age- and sex-adjusted 30 patients, a pair of 15 patients with and without SPIP with tolerance factor 0, were selected for the study of chemerin, YKL-40, resistin, and IL6. Continuous end-points at different time points between 2 independent groups were compared using the non-parametric Mann-Whitney test, for the age- and sex-matched patients using the Wilcoxon signed-rank test, and the Kruskal-Wallis test for the 3 time points within each group.

Statistical power was calculated. Analyses were performed with IBM SPSS Statistics version 28.0 (IBM Corporation, Armonk, NY, USA) with *P *< 0.05 (2-tailed) as the significance criterion.

## RESULTS

### Patient characteristics

A total of 74 patients were included in the study, 37 patients with and 37 without wound analgesia. None of the patients had immunosuppressive treatment such as corticosteroids. Patients with SPIP were older as compared to those without (66.7 [10.7] vs 60.2 [13.4], years, respectively, *P *= 0.045). None of the patients had connective tissue disorder, vasculitis, or previous cardiac surgery (Supplementary Table S1). Preoperative lung functions, including FEV, FVC, and PEF, did not differ between the patient groups (Supplementary Table S2). Neither patient characteristics nor preoperative lung functions differed among the matched patient groups (Supplementary Tables S1 and S2).

### Postoperative details

Results for postoperative lung functions are shown in **Table 1**. FEV change, FVC change, and Relative FVC change decreased less in patients treated with SPIP as compared to those without (*P *= 0.024, *P *= 0.042, and *P *= 0.042). For the effect size of −1.8 (0.9) vs −2.4 (0.9), with SD 0.9, with patient numbers 37 and 37, with alpha 0.05, and 2-tailed, the statistical power was 81.8% for FVC change indicating adequate statistical power. There were no differences in total oxycodone use per body mass index (BMI) in patients with and without SPIP (3.7 [1.3] and 4.1 [1.6], *P *= 0.634, respectively). There were no differences in postoperative lung functions between all patients versus age-and sex-matched patients. For the age- and sex-matched patients, PEF decreased less in patients treated with SPIP as compared to those without (*P *= 0.048).

**Table 1.** Postoperative Lung Functions

Variable	All patients	With SPIP	Without SPIP	*P*-value	Matched patients*	With SPIP	Without SPIP	*P*-value
**Number**	74	37	37	>0.99	30	15	15	>0.99
**FEV (L)**	1.4 (0.5)	1.5 (0.5)	1.3 (0.4)	0.137	1.5 (0.6)	1.6 (0.6)	1.4 (0.5)	0.363
**FVC (L)**	1.6 (0.6)	1.7 (0.7)	1.5 (0.5)	0.316	1.7 (0.7)	1.9 (0.8)	1.6 (0.6)	0.451
**PEF (L/s)**	233.6 (95.1)	255.7 (106.0)	212.17 (78.8)	0.071	257.9 (106.7)	300.8 (112.2)	212.0 (81.1)	0.048
**FEV change (L)**	−1.5 (0.7)	−1.3 (0.7)	−1.7 (0.7)	0.024	−1.6 (0.7)	−1.4 (0.9)	−1.9 (0.4)	0.465
**FVC change (L)**	−2.1 (0.9)	−1.8 (0.9)	−2.4 (0.9)	0.042	−2.2 (0.9)	−2.0 (1.1)	−2.5 (0.5)	0.273
**PEF change (L/s)**	−272.8 (163.8)	−259.9 (194.6)	−293.3 (102.5)	0.174	−227.0 (135.4)	−147.4 (66.6)	−366.3 (107.6)	0.180
**Relative FEV change**	−0.5 (0.2)	−0.5 (0.2)	−0.6 (0.1)	0.053	−0.5 (0.2)	−0.5 (0.3)	−0.6 (0.1)	0.715
**Relative FVC change**	−0.6 (0.2)	−0.5 (0.2)	−0.6 (0.1)	0.042	−0.6 (0.2)	−0.5 (0.2)	−0.7 (0.1)	0.273
**Relative PEF change**	−0.5 (0.2)	−0.5 (0.2)	−0.6 (0.1)	0.089	−0.4 (0.2)	−0.3 (0.1)	−0.7 (0.2)	0.180

Values represent means (standard deviation). *: matched for age and sex; FEV: forced expiratory volume in 1 second; FEV change: preoperative FEV minus postoperative FEV; FVC: forced vital capacity; FVC change: preoperative FVC minus postoperative FVC; PEF: peak expiratory flow; PEF change: preoperative PEF minus postoperative PEF; Relative FEV change: preoperative FEV minus postoperative FEV divided by preoperative FEV; Relative FVC change: preoperative FVC minus postoperative FVC divided by preoperative FEV; Relative PEF change: preoperative PEF minus postoperative PEF divided by preoperative PEF; SPIP: ultrasound-guided superficial parasternal intercostal plane block.

### Levels of chemerin, YKL-40, resistin, and IL6

There were no differences at any time points T1, T2, or T3 in the levels of either venous or arterial chemerin, YKL-40, resistin, and IL6 between patients with vs without SPIP (Supplementary Tables S3 and S4). The decrease in arterial chemerin from T1 to T3 was significant in patients with SPIP as compared to patients without (125.1 [29.1] to 95.4 [23.9], *P *= 0.004 vs 131.6 [33.6] to 108.3 [38.3], *P *= 0.073, respectively).

### Lung filtration capacity

**Figure 1.**
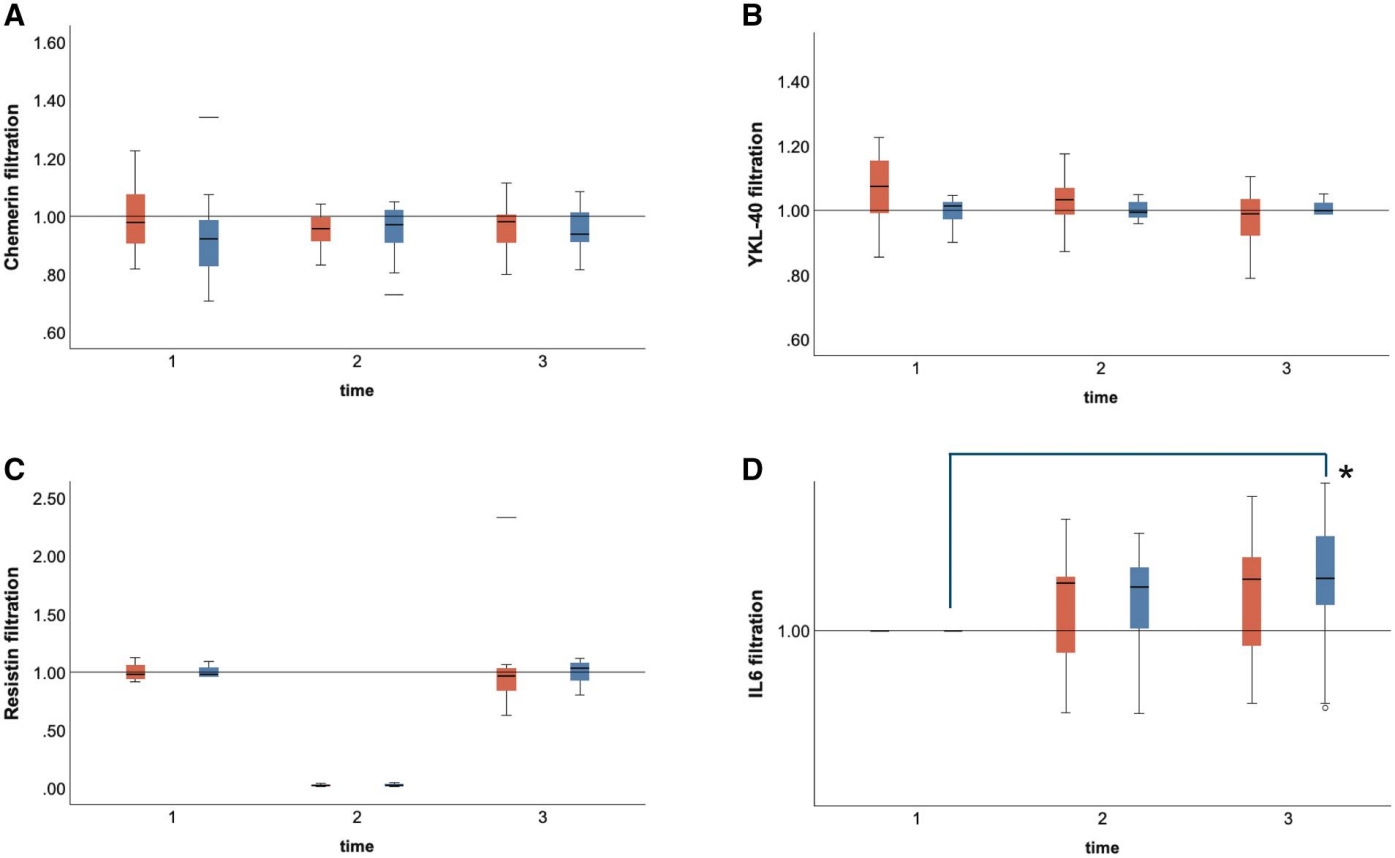
Calculated Ratio of the Venous Chemerin, YKL-40, Resistin, and Interleukin-6 (IL6) Divided By the Simultaneous Arterial Chemerin (A), YKL-40 (B), Resistin (C), and IL6 (D) Immediately Before Surgery (1), At Surgery (2), and Postoperatively the Day After Surgery (3) in Patients Undergoing Aortic Valve Replacement Via Full Sternotomy with SPIP (Blue Bars) and Without SPIP (Red Bars). Horizontal lines of the box show the median. Lines above and below the box indicate the 75th and 25th percentiles, respectively. Note increased venous/artery IL6 ratio > 1 in patients with SPIP at T3 versus T1, indicating excessive filtration of proinflammatory IL6 (D). *P = 0.024. YKL-40: chitinase-3-like protein 1.

Since individual blood values alone do not reflect their passage through the lungs, we measured the simultaneous venous and arterial values of chemerin, YKL-40, resistin, and IL6. The values representing lung filtration capacity, venous/arterial chemerin, venous/arterial YKL-40, and venous/arterial resistin ratios did not change from time points T1, T2, and T3 in the patients with vs without SPIP (**Figure 1**). However, there was a significant increase in venous/arterial IL6 ratio in patients with SPIP as compared with those without from time points T1, T2, and T3 (1.0 [0.2], 1.1 [0.2], and 1.2 [0.2], *P *= 0.024 vs 1.6 [2.3], 1.1 [0.3], and 1.1 [0.2], *P *= 0.332, respectively).

## DISCUSSION

This study shows that SPIP in patients undergoing aortic valve replacement via full sternotomy is associated with early restoration of postoperative lung function and filtration capacity of the inflammatory IL6 cytokine.

Lung function tests add to the objective evaluation of recovery after surgery.^16^ As expected, lung functions decrease in all patients after full sternotomy and aortic valve replacement. Decreased postoperative lung function may reflect excessive postoperative pain, especially in our patients without additional local wound analgesia as compared with those with SPIP. It is tempting to suggest that additional SPIP in patients undergoing full sternotomy and aortic valve replacement is associated with ameliorated postoperative lung functions. Indeed, pain control may improve early mobility and aids early lung recovery. In our study, FEV and FVC changes after surgery decreased less in patients with SPIP versus without. These variables may serve as markers for early restoration of lung function capacity after aortic valve replacement and sternotomy as statistical power was up to 81.8%. In addition, early restoration of lung function, as observed with PEF, was also noted in the age- and sex-matched patients with SPIP versus without.

To the best of our knowledge, the association of markers of inflammation with lung filtration capacity has not been investigated in the acute setting of recovery after full sternotomy and cardiac surgery. Enhanced lung function may ameliorate lung filtration capacity. The net serum concentrations of chemerin, YKL-40, resistin, and IL6 were estimated by comparing their venous/arterial ratios between patients with versus without SPIP.^15^ As being in between these 2 samples, it was investigated whether the pulmonary vasculature participated in filtrating these markers of inflammation upon lung function capacity.^28^ With the aid of calculating the simultaneous venous/arterial ratio during surgery, it was, however, demonstrated that the concentrations of chemerin, YKL-40, and resistin did not alter during their passage through the lungs in patients either with or without SPIP. On the other hand, in patients with SPIP, venous/arterial IL6 ratios increased from time point T1 to T3, indicating that excessive IL6 was filtrated in the lungs as compared to patients without wound analgesia. As a ubiquitous inflammatory cytokine, IL6 may represent an end-stage inflammatory marker that reflects lung dysfunction after cardiac surgery.^29^

Chemerin and its receptor are expressed in various inflammatory cells, as well as in neurons and glia cells in the dorsal root ganglion, spinal cord, and retina.^30^^,^^31^ In animal models of asthma and pain, nociception was associated with chemerin.^20^ The common denominator of these experimental models may be linked to inflammation in general, a well-known early reaction also after cardiac surgery, least to mention full sternotomy. A chemerin-associated potent inhibitor of airway inflammation and neuropathic pain has already been envisaged based on a translational study.^30^ We observed a more prominent decreased of plasma chemerin levels after surgery in patients with SPIP as compared to those without despite similar consumption of oxycodone.

Besides chemerin, the adipose tissue as an endocrine organ secretes resistin that influences the release of inflammatory mediators and impacts lung functions.^32^ Resistin is a proinflammatory adipokine that may induce angiogenesis, and smooth muscle cell proliferation. Since some adipokines interact with each other, not only the total concentrations but also the balance of pro- and anti-inflammatory adipokines may be important.^33^ Increased resistin levels were observed in all patients after aortic valve replacement and full sternotomy.

Increased serum YKL-40 may indicate excessive cleavage of the glycoprotein-like molecule on the endothelium of the arteries, suggesting an increased activation of proinflammatory and profibrotic cytokines after ischaemia due to the surgical trauma.^26^ YKL-40 reflects tissue remodelling after ischaemia.^34^ Increased YKL-40 and IL6 reflect tissue injury after cardiopulmonary bypass and enhance macrophage activation,^35^ whereas adipokines, such as chemerin and resistin, may act as regulators of macrophage function.^36^ Taken together, macrophages may have a common role in mediating the effect of chemerin, resistin, YKL-40 and IL6. Both YKL-40 and IL6 were increased in all patients after surgery in this study.

It is, however, beyond the scope of this study to speculate on exact mechanisms involved during lung filtration. Although chemerin may participate in the process of vascular remodelling through aortic smooth muscle cell proliferation and migration during the development of hypertension, these mechanisms may rather explain chronic involvement of inflammation and trauma.^20^ Indeed, cardiac surgery includes a relatively acute and short-standing inflammation due to surgical trauma including the wound.^15^ As a potential marker of subsiding inflammation, chemerin was decreased in our patients with SPIP, again associating pain control with early recovery and lung function. The mediators of inflammation in this study reflect the lung function capacity after cardiac surgery.

The study included a relatively small number of patients with strict inclusion and exclusion criteria; the careful selection of study patients took up to 5 years, and most recent patients with hemisternotomy were excluded from the study. The inflammatory responses were investigated in only 30 age- and sex-adjusted patients. However, SPIP immediately before surgical aortic valve replacement via full sternotomy has a major net impact on postoperative lung function. Estimating IL6 lung passage with the venous/arterial ratio may provide a practical means for the evaluation of pain control and lung function after cardiac surgery; it remains to be shown whether SPIP and associated less severe impairment of lung function may affect long-term clinical outcome after aortic valve replacement and full sternotomy.

## CONCLUSION

We identified increased IL6 lung filtration and restoration of lung function in patients with SPIP undergoing aortic valve replacement via full sternotomy. Further investigation of the impact of SPIP and lung function in patients undergoing cardiac surgery may be anticipated.

## AUTHOR CONTRIBUTIONS

The authors comply with the role of professional medical writers and the criteria recommended by the International Committee of Medical Journal Editors.

Conceptualization (E.M., K.J., A.M.), data curation (A.K., J.V., A.M.), formal analysis (A.K., K.J., E.M., A.M.), funding acquisition (A.K., K.J., A.M.), investigation (A.K., J.V., E.M., K.J., A.M.), methodology (A.K., J.V., E.M., K.J., A.M.), project administration (E.M., K.J., A.M.), software (A.K., J.V., E.M., K.J., A.M.), resources (A.K., E.M., K.J., A.M.), supervision (K.J., A.M.), validation (A.K., J.V., E.M., K.J., A.M.), visualization (A.M.), writing-original draft (A.K., A.M.), and writing-review and editing (A.K., J.V., E.M., K.J., A.M.).

## SUPPLEMENTARY MATERIAL

Supplementary material is available at *EJCTS* online.

## FUNDING

This work was supported by research funding from the Competitive State Research Financing of the Expert Responsibility Area of Tampere University Hospital, Tampere Tuberculosis Foundation, The Finnish Cultural Foundation, and Sohlberg Foundation.

## CONFLICTS OF INTEREST

None declared.

## DATA AVAILABILITY

The data in this study will be provided upon reasonable request to the corresponding author after approval from the Tampere University Hospital Ethics Committee.

## Supplementary Material

ezaf226_Supplementary_Data
