# Supplementary material for: Superficial Parasternal Intercostal Plane Block and Full Sternotomy; A Randomized Trial
Source: Eur J Cardiothorac Surg. 2025 Jul 8;67(7):ezaf226. doi: 10.1093/ejcts/ezaf226 (PMC12282944; doi:10.1093/ejcts/ezaf226)
Supplement: ezaf226_Supplementary_Data [file ezaf226_supplementary_data.zip › SupplTablesClean.docx]

**Supplementary table 1.** Patient characteristics.

| Variable | All patients | With  SPIP | Without SPIP | *P*-value | Matched patients* | With  SPIP | without SPIP | *P*-value | |
| --- | --- | --- | --- | --- | --- | --- | --- | --- | --- |
|  |  |  |  |  |  |  |  | |  |
| Number | 74 | 37 | 37 | >0.99 | 30 | 15 | 15 | | >0.99 |
| Age, mean (SD) | 63.4 (12.5) | 66.7 (10.7) | 60.2 (13.4) | 0.045 | 63.5 (9.9) | 63.5 (10.1) | 63.5 (10.1) | | >0.99 |
| Female, n | 53 | 26 | 27 | >0.99 | 28 | 14 | 14 | | >0.99 |
| BMI, mean (SD) | 28.5 (5.1) | 27.3 (4.2) | 29.7 (5.7) | 0.067 | 29.3 (5.4) | 28.2 (4.4) | 30.4 (6.2) | | 0.351 |
| Hypertension, n | 38 | 18 | 20 | 0.816 | 16 | 6 | 10 | | 0.272 |
| Dyslipidemia, n | 13 | 8 | 21 | 0.302 | 9 | 6 | 3 | | 0.427 |
| Diabetes, n | 11 | 7 | 4 | 0.515 | 5 | 3 | 2 | | >0.99 |

SPIP = Ultrasound-guided superficial parasternal intercostal plane block

*= matched for age and sex; SD= standard deviation; BMI= body mass index

**Supplementary table 2.** Preoperative lung functions

| Variable | All patients | With  SPIP | Without SPIP | *P*-value | Matched patients* | With  SPIP | Without SPIP | *P*-value |
| --- | --- | --- | --- | --- | --- | --- | --- | --- |
|  |  |  |  |  |  |  |  |  |
| Number | 74 | 37 | 37 | >0.99 | 30 | 15 | 15 | >0.99 |
| FEV (L) | 2.8 (0.8) | 2.7 (0.8) | 3.0 (0.9) | 0.167 | 3.0 (0.6) | 2.9 (0.7) | 3.0 (0.5) | 0.773 |
| FVC (L) | 3.6 (1.1) | 3.5 (1.0) | 3.8 (1.2) | 0.316 | 3.8 (0.8) | 3.8 (1.0) | 3.8 (0.6) | 0.715 |
| PEF (L/s) | 503.4 (149.9) | 510.3 (179.0) | 492.5 (92.8) | 0.903 | 504.8 (66.3) | 480.7 (61.3) | 547.0 (58.3) | 0.180 |

SPIP = Ultrasound-guided superficial parasternal intercostal plane block

*= matched for age and sex, FEV= forced expiratory volume in one second; Values represent means (standard deviation); FVC= forced vital capacity; PEF= peak expiratory flow

**Supplementary table 3**. Perioperative and postoperative venous chemerin, YKL-40, resistin and IL6 in patients undergoing aortic valve surgery through full sternotomy. Mean (standard deviation)

| Sample time | Variable | With  SPIP (n=15) | Without  SPIP (n=15) | *P*-value |
| --- | --- | --- | --- | --- |
|  |  |  |  |  |
| After induction of anesthesia: T1 | chemerin | 118.4 (41.2) | 137.2 (55.8) | 0.427 |
|  | YKL-40 | 55.6 (47.9) | 89.6 (141.2) | 0.570 |
|  | resistin | 21.7 (6.7) | 25.2 (9.2) | 0.281 |
|  | IL6 | 1.4 (1.1) | 1.6 (2.3) | >0.99 |
| 15 min after releasing aortic cross clamp: T2 | chemerin | 97.9 (36.9) | 103.0 (32.6) | 0.394 |
|  | YKL-40 | 65.2 (53.9) | 104.3 (182.0) | 0.733 |
|  | resistin | 49.4 (23.3) | 43.3 (12.8) | 0.865 |
|  | IL6 | 50.5 (22.7) | 59.8 (29.9) | 0.826 |
| 20 hours after releasing aortic cross clamp: T3 | chemerin | 91.5 (24.2) | 104.7 (37.7) | 0.233 |
|  | YKL-40 | 1277.8 (1100.9) | 2651.9 (5147.5) | 0.496 |
|  | resistin | 49.4 (23.3) | 54.3 (23.5) | 0.609 |
|  | IL6 | 50.4 (22.7) | 61.8 (29.7) | 0.198 |

SPIP = Ultrasound-guided superficial parasternal intercostal plane block

IL6= interleukin-6

**Supplementary table 4**. Perioperative and postoperative arterial chemerin, YKL-40, resistin and IL6 in patients undergoing aortic valve surgery through full sternotomy. Mean (standard deviation)

| Sample time | Variable | With  SPIP (n=15) | Without  SPIP (n=15) | *P*-value |
| --- | --- | --- | --- | --- |
|  |  |  |  |  |
| After induction of anesthesia: T1 | chemerin | 125.1 (29.1) | 131.6 (33.6) | 0.334 |
|  | YKL-40 | 56.1 (49.1) | 85.9 (138.9) | 0.570 |
|  | resistin | 26.2 (17.4) | 26.9 (11.3) | 0.394 |
|  | IL6 | 1.6 (1.7) | 1.0 (0) | 0.180 |
| 15 min after releasing aortic cross clamp: T2 | chemerin | 107.2 (54.9) | 108.3 (33.1) | 0.334 |
|  | YKL-40 | 64.8 (53.2) | 104.9 (193.4) | 0.955 |
|  | resistin | 41.1 (16.6) | 44.9 (12.9) | 0.427 |
|  | IL6 | 68.5 (68.7) | 54.2 (24.3) | 0.683 |
| 20 hours after releasing aortic cross clamp: T3 | chemerin | 95.4 (23.9) | 108.3 (38.3) | 0.363 |
|  | YKL-40 | 1278.6 (1168.8) | 1489.5 (1354.3) | 0.910 |
|  | resistin | 49.2 (31.5) | 53.9 (26.7) | 0.496 |
|  | IL6 | 41.9 (16.5) | 54.6 (26.6) | 0.084 |

SPIP = Ultrasound-guided superficial parasternal intercostal plane block

IL6= interleukin-6
