# Supplementary material for: Superficial Parasternal Intercostal Plane Block and Full Sternotomy; A Randomized Trial
Source: Eur J Cardiothorac Surg. 2025 Jul 8;67(7):ezaf226. doi: 10.1093/ejcts/ezaf226 (PMC12282944; doi:10.1093/ejcts/ezaf226)
Supplement: ezaf226_Supplementary_Data [file ezaf226_supplementary_data.zip › SupplemFileClean2.docx]

**Supplementary file.**

**Ethical Statement and study design**

The study was approved by the Tampere University Hospital Ethics Committee (R18011M, 2/6/2018) complying with the principles laid down in the Declaration of Helsinki (Recommendations guiding physicians in biomedical research involving human subjects, adopted by the 18^th^ World Medical Assembly, Helsinki, Finland, June 1964), and all patients gave written consent. We included patients aged 18 to 85 years, undergoing elective aortic valve replacement surgery using standard medial sternotomy. Exclusion criteria were diabetes mellitus with complications, limited proficiency in Finnish, preoperative use of painkillers or antidepressants, and chronic pain.

Wound analgesia was provided with a single 20 ml injection per side using the SPIP technique [27] by two anesthetists (A.K. and K.J.) or under their supervision by the attending anesthetist; briefly, the injection was performed in between the third and the fourth rib, below the pectoralis major and above the external intercostal muscles, on both sides of the sternum. Postoperative incisional analgesia consisted of a per oral long-acting oxycodone and intravenous oxycodone on demand and was assessed by oxycodone consumption until day 1 after surgery and expressed as mg x m^2^/kg.

A research assistant organized block randomization utilizing blocks of four patients. The patient's group allocations were typed on separate pages, folded, and concealed in sequentially numbered sealed opaque envelopes. The envelopes were then opened by the responsible anaesthesia nurse for each patient. The anaesthesia nurse prepared two 20ml syringes with the study drug without a label. Patients randomized to receive SPIP received 40mls of 7,5mg/ml ropivacaine (Ropivacaine Orion 7,5mg/ml), and others received 40mls of saline only (Natriumklorid Fresenius Kabi 9mg/ml). The surgeon, attending anesthetist, and nurses working in the ICU were not informed of the group assignment.


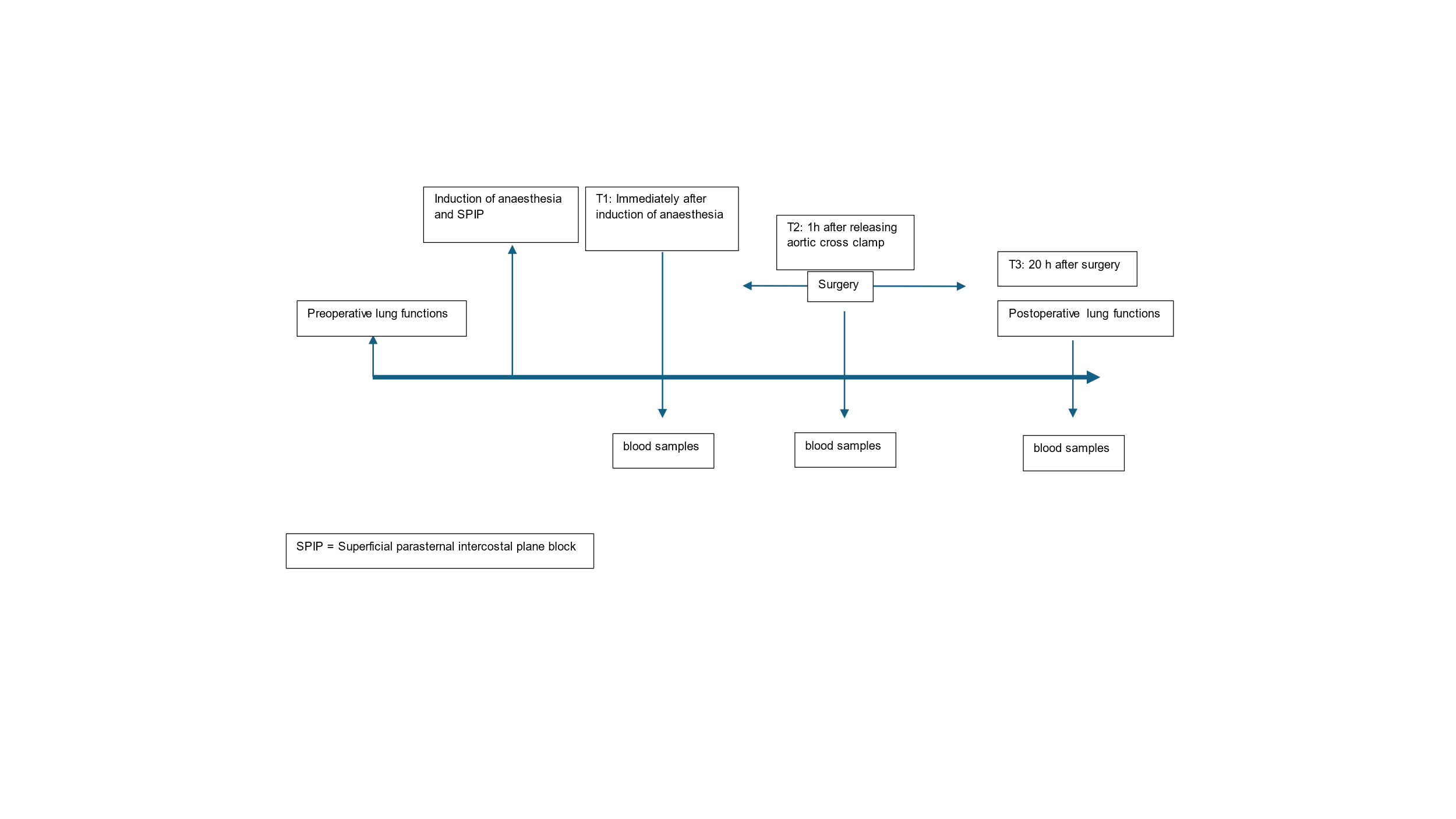
**Timeline diagram**
